# Impact of Recommended Maternal Vaccination Programs on the Clinical Presentation of SARS-CoV-2 Infection: A Prospective Observational Study

**DOI:** 10.3390/vaccines9010031

**Published:** 2021-01-08

**Authors:** Maria Luisa de la Cruz Conty, Maria Begoña Encinas Pardilla, Marta Garcia Sanchez, Laura Gonzalez Rodriguez, Marta Luisa Muner-Hernando, Ana Royuela Vicente, Pilar Pintado Recarte, Alicia Martinez Varea, Clara Martinez Diago, Sara Cruz Melguizo, Oscar Martinez-Perez, on behalf of the Spanish Obstetric Emergency Group

**Affiliations:** 1 Fundacion de Investigacion Biomedica, Puerta de Hierro University Hospital of Majadahonda, 28222 Madrid, Spain; farmcruz@gmail.com; 2 Department of Gynecology and Obstetrics, Puerta de Hierro University Hospital of Majadahonda, 28222 Madrid, Spain; mariabegona.encinas@salud.madrid.org (M.B.E.P.) ; scruzm@salud.madrid.org (S.C.M.); omartinez@ucam.edu (O.M.-P.); 3 Department of Gynecology and Obstetrics, Quironsalud Malaga University Hospital, 29004 Malaga, Spain; 4 Department of Gynecology and Obstetrics, Alvaro Cunqueiro Hospital of Vigo, 36213 Pontevedra, Spain; Laura.Gonzalez.Rodriguez@sergas.es; 5 Department of Gynecology and Obstetrics, La Paz University Hospital, 28046 Madrid, Spain; martal.muner@salud.madrid.org; 6 *Biostatistics Unit, Puerta de Hierro Biomedical Research Institute (IDIPHISA-CIBERESP), 28222 Madrid, Spain; aroyuela@idiphim.org; 7 Department of Gynecology and Obstetrics, Gregorio Marañon University Hospital, 28007 Madrid, Spain; ppintado@salud.madrid.org; 8 Department of Gynecology and Obstetrics, La Fe University and Polytechnic Hospital, 46026 Valencia, Spain; martinez_alivar@gva.es; 9 Department of Gynecology and Obstetrics, Doctor Josep Trueta University Hospital of Girona, 17007 Girona, Spain; clmartinez.girona.ics@gencat.cat; 10 Departamento de Obstetricia y Ginecología, Universidad Autónoma de Madrid, 28029 Madrid, Spain

**Keywords:** SARS-CoV-2, covid 19, pregnancy, passive immunization, maternal immunization, influenza vaccines, diphtheria tetanus pertussis vaccine

## Abstract

The COVID-19 pandemic has raised questions about the possible cross immunity resulting from common vaccination programs and SARS-CoV-2 infection. Therefore, the Spanish Obstetric Emergency group performed a multicenter prospective study on the vaccination status of Influenza and Tdap (diphtheria, tetanus and pertussis vaccine boost administered in adulthood) in consecutive cases of SARS-CoV-2 infection in a pregnancy cohort, in order to assess its possible association with the clinical presentation and severity of symptoms of SARS-CoV-2 infection, as well as to determine the factors that may affect vaccination adherence. A total of 1150 SARS-CoV-2 positive pregnant women from 78 Spanish hospitals were analyzed: 183 had not received either vaccine, 23 had been vaccinated for Influenza only, 529 for Tdap only and 415 received both vaccines. No association was observed between the vaccination status and the clinical presentation of SARS-CoV-2 infection and/or the severity of symptoms. However, a lower adherence to the administration of both vaccines was observed in the Latin-American subgroup. Based on the results above, we reinforce the importance of maternal vaccination programs in the actual pandemic. Health education campaigns should be specially targeted to groups less likely to participate in these programs, as well as for a future SARS-CoV-2 vaccination campaign.

## 1. Introduction

The COVID-19 pandemic has raised questions among the scientific community about the possible cross immunity resulting from common vaccination programs and SARS-CoV-2 infection. Although evidence at the molecular level has not been provided yet, there are studies that report an association between Influenza vaccination and a lower risk of serious illness and/or death among COVID-19 patients [1,2,3,4]. In addition, a lower rate of Influenza vaccination has been observed among COVID-19 patients requiring hospitalization, intensive care or respiratory support, and an inversely proportional association was also found between Influenza vaccination and mortality risk in these patients [5]. 

It has also been suggested that the DTP vaccine (a combination of vaccines against diphtheria, tetanus and pertussis) could confer potential cross-reactivity to SARS-CoV-2 due to the existence of peptide matches between both, though clinical trials and/or broad observational studies are needed to confirm this hypothesis built on molecular findings [6]. 

On the other hand, the theoretical associations above can be affected by multiple factors that must be studied, such as ethnicity. It seems that the progression of COVID-19 is worse in people of certain ethnicities, with an increase in Intensive Care Unit (ICU) admission of Hispanic and non-Hispanic black pregnant women [7,8]; in turn, it should be considered that the adherence to vaccination programs varies according to ethnicity, even in countries with a national public health system.

Based on these theories, and as pregnancy is an exceptional period in adulthood in which Influenza and DTP vaccines are administered coincidentally or closely, we performed an observational prospective study in approximately 1400 SARS-CoV-2 positive pregnant women diagnosed from 26 February to 5 November 2020 in 78 Spanish hospitals. The objective was to assess the possible association between the clinical presentation and severity of symptoms of SARS-CoV-2 infection with their Influenza and DTP vaccination status. Moreover, we examined the factors that may affect Influenza and DTP vaccination adherence, taking into account that in Spain, with a public and universal health system, these vaccines are accessible and free for the population and strongly recommended for pregnant women. Finally, the possible relationship between these factors and the clinical presentation of the SARS-CoV-2 infection was also analyzed. 

## 2. Materials and Methods

This was a multicenter prospective study of consecutive cases of SARS-CoV-2 infection in a pregnancy cohort registered by the Spanish Obstetric Emergency group [9]. The registry protocol was approved by the coordinating hospital’s Medical Ethics Committee on 23 March 2020 (reference number: PI 55/20) and each collaborating center subsequently obtained protocol approval locally; the registry protocol is available in ClinicalTrials.gov (NCT04558996). A complete list of the 78 centers contributing to the study is provided in Table S1. Upon recruitment, given the contagiousness of the disease and the lack of personal protection equipment, mothers consented by either signing a document, when possible, or by giving permission verbally, which was recorded in the patient’s chart. A specific database was designed for recording information regarding SARS-CoV-2 infection in pregnancy and the data were entered by the lead researcher for each center after delivery.

The Influenza and DTP vaccinations are included in the Spanish protocol for care and control of pregnancies [10,11] and both vaccines are free and very accessible to pregnant women through the National Health Care System. The DTP vaccine used is the Tdap, a DTP vaccine boost administered in adulthood (Boostrix^®^).

### 2.1. Infected Cohort

During the period of the study, from 26 February to 5 November 2020, we selected COVID-19 obstetric patients detected by screening for SARS-CoV-2 infection at admission in the delivery ward, or by testing suspicious cases that came into hospital due to COVID-19 symptoms. SARS-CoV-2 infection was diagnosed by positive double-sampling polymerase-chain-reaction (PCR) from nasopharyngeal swabs. All positive cases with known Influenza and Tdap vaccination status were included in the study. The cases were classified as asymptomatic and symptomatic, and the latter was stratified into three groups: mild–moderate symptoms, pneumonia and complicated pneumonia/shock (with ICU admission and/or mechanical ventilation and/or septic shock) [12].

Information regarding the demographic characteristics of each pregnant woman, Influenza and Tdap vaccination, comorbidities, previous and current obstetric history were extracted from the clinical and verbal history of the patient. 

### 2.2. Statistical Analysis

The variable maternal age (years) was tested for normal distribution using the Kolmogorov–Smirnov test. Descriptive data are presented as median (interquartile range, IQR) or number (percentage). *p*-values were obtained by the Kruskal–Wallis test and post-hoc pairwise comparison for the numerical variable and Pearson Chi-squared test for categorical variables. A *p*-value below 0.05 was considered statistically significant. In case of a statistically significant association between Influenza and/or Tdap vaccinations and the clinical presentation of SARS-CoV-2 infection, the potential influence of known and suspected measured confounding factors was controlled with multivariable logistic and multinomial regression modeling, after checking scientifically sound two-way interactions. 

Data were analyzed using SPSS version 20 (IBM Inc., Chicago, IL, USA) and the lme4 package in R, version 3.4 (RCore Team, 2017) [13].

## 3. Results

### 3.1. Description of the Infected Cohort According Their Vaccination

**Figure 1:**
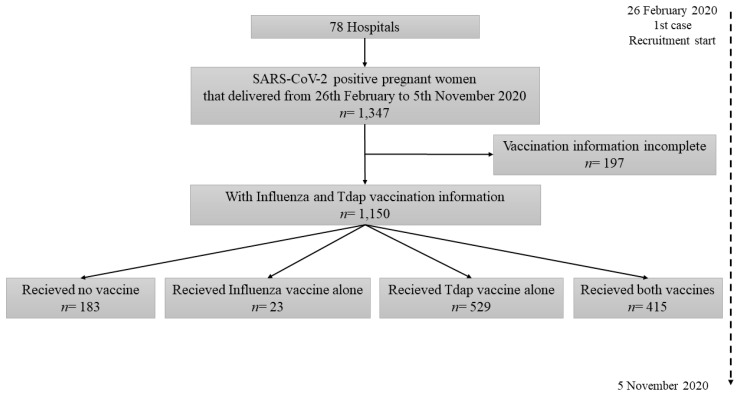
Flow chart of the study data.

One thousand three hundred and forty-seven positive SARS CoV-2 pregnant women were identified, of which 197 were excluded because they did not provide complete information of their Influenza and/or Tdap vaccination status. Thus, a total of one thousand hundred fifty (1150) patients were analyzed: 183 had not received either vaccine, 23 had been vaccinated for Influenza only, 529 for Tdap only and 415 received both vaccines (Figure 1).

**Figure 2:**
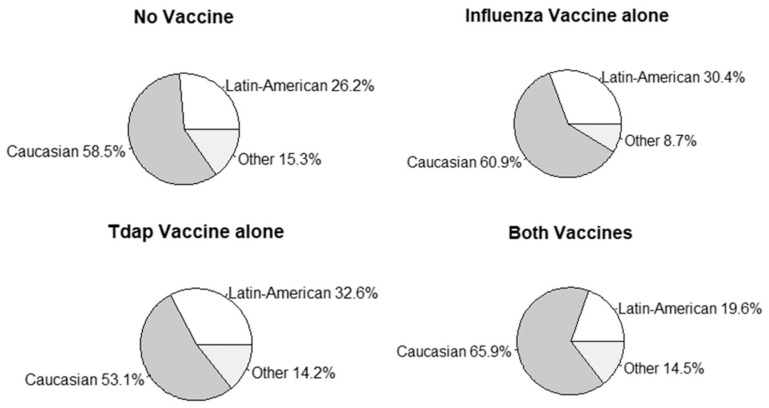
Ethnicity distribution by vaccination group.

Table 1 shows the baseline characteristics of patients stratified into the four vaccination groups mentioned above. Pregnant women vaccinated for both Influenza and Tdap significantly differed from other groups: these women were older (*p* = 0.013), the proportion of Latin-Americans in this group was significantly lower (19.6% vs. above 26.0% in the remaining vaccination groups, *p* = 0.001, Figure 2) and in vitro fertilization was significantly more frequent among these patients (8.7% vs. below 5.0% in other groups, *p* = 0.025). Private hospital attendance and parity showed no significant differences between groups, while the proportion of pregnant women with respiratory comorbidities in the Influenza vaccine group quadrupled the observed in the remaining groups (17.4% vs. below 4.5% in the remaining vaccination groups, *p* = 0.007).

On the other hand, and independently of the vaccination status of patients, an association between the type of hospital and ethnicity was observed, being private hospital attendance five times more frequent in Caucasian women (10.8% vs. 2.1% in other ethnicities, *p* < 0.001).

### 3.2. Clinical Presentation of SARS-CoV-2 Infection According to the Vaccination of Patients

Clinical presentation of SARS-CoV-2 infection is shown in Table 2. The observed distribution of asymptomatic and symptomatic patients was similar between vaccination groups (approximately 50% vs. 50%), with the exception of those patients vaccinated only for Influenza (26.1% vs. 73.9%, respectively); even so, this difference was not statistically significant (*p* = 0.051). When analyzed by clinical presentation, approximately three quarters of symptomatic patients had mild–moderate symptoms (cough, anosmia, fatigue/discomfort, fever, dyspnea, etc.) in the groups that received both vaccines, Tdap vaccine alone and no vaccine at all, while one quarter of symptomatic pregnant women developed more severe symptoms (pneumonia with/without ICU admission, mechanical ventilation and/or septic shock). However, the distribution of symptoms was slightly different in the group vaccinated only for Influenza, where the proportion of symptomatic patients who developed pneumonia or complicated pneumonia/shock increased up to 41.2% although, as in the previous case, these differences were not statistically significant (*p* = 0.433).

### 3.3. Baseline Characteristics of Asymptomatic and Symptomatic Patients

Due to the absence of a statistically significant association of vaccination with the clinical presentation of SARS-CoV-2 infection, no multivariable analysis was carried out, but Table 3 was constructed to study baseline characteristics of asymptomatic and symptomatic patients that could confound the findings above. It was observed that among the symptomatic group there were twice as many patients with respiratory comorbidities than in the asymptomatic group (5.1% vs. 2.6%, respectively, *p* = 0.024) and more women from Latin-America (35.8% vs. 18.2% in asymptomatic patients, *p* < 0.001), as well as the proportion of Latin-American women increased up to 45.2% among patients with pneumonia or complicated pneumonia/shock (*p* = 0.032). Additionally, the use of private health care was less common among positive pregnant women who developed COVID-19 symptomatology (4.4% vs. 9.9% of asymptomatic patients, *p* < 0.001), characteristic in turn associated with ethnicity, as mentioned above.

## 4. Discussion

The study we provide is part of one of the largest worldwide databases of pregnancy and SARS-CoV-2 with 78 hospitals involved and almost 1400 deliveries from infected pregnant women included (from 26th February to 5th November 2020, therefore, including cases of both the first and second COVID-19 wave), whether they were symptomatic or not. The main strength of this study is that the patients came from all over Spain, with their inherent demographic and healthcare differences, and they were users of public and private hospitals.

Our patients represent a unique population in terms of immunology and vaccination status, due to the immunomodulation variations that characterize pregnancies and it corresponds to an exceptional period in adulthood in which Influenza and Tdap vaccines are administered coincidentally or closely, except for some specific immunodeficiency situations. This represents the uniqueness of this study; a pioneer in analyzing the adherence to the recommended vaccines during pregnancy and the factors that may influence this adherence, as well as analyzing the possible relationship between them and the clinical presentation of the SARS-CoV-2 infection.

Vaccination against Influenza and pertussis in pregnancy is a recommendation supported by the WHO and is already applied in many countries, but despite the evidence demonstrated on safety and effectiveness, vaccination adherence is still moderate for pertussis and low for Influenza in pregnant women [14]. Spain has achieved high rates of coverage, although there exist clear differences between Tdap and Influenza (80.1% vs. 40.6% for the 2018–2019 campaign in pregnant women) [15]. In our study of SARS-CoV-2 positive patients, we found similar rates of vaccine compliance (82.1% and 38.1% for Tdap and Influenza vaccination, respectively), which confirms the representativeness of our participants and confers robustness of our study results.

When the vaccination analysis was stratified by ethnicity, a lower adherence to the administration of both vaccines was observed in the Latin-American subgroup; this coincides with the trend published in other countries for vaccination of racial minorities [16]. It seems that the observed patient profile who tends to complete the double vaccination in Spain corresponds to older Caucasian women, many of whom required in vitro fertilization techniques, a fact that may as well justify the higher frequency of private health care services attendance in this group and an increased awareness of the importance of their vaccination due to risk factors characteristic of in vitro fertilization users. These differences cannot be attributed in our case to the ease of access to the vaccines or their cost, since in Spain these two vaccines are free of cost and standardized for pregnant women throughout the country. Therefore, there are other factors such as cultural, beliefs, fears, health education, that come into play, which have not been the object of this study [17,18].

No association was observed between the vaccination status of patients in the current pregnancy (no vaccination, Influenza or Tdap vaccine and double vaccination) and the clinical presentation of SARS-CoV-2 infection and/or the severity of symptoms (development of pneumonia, its complication with ICU admission or need of mechanical ventilation or septic shock), as previously reported by Martínez-Baz in a cohort of Health Workers [19]. Still, it should be highlighted that symptomatic patients are over-represented in our study population since not all participating hospitals had a universal antenatal screening program for SARS-CoV-2 infection (so only identified symptomatic cases by passive surveillance) or implemented the program later.

On the other hand, and unlike Tdap and Influenza vaccines (both inactivated vaccines), it has been suggested the potential effect of live attenuated vaccines such as the Bacille Calmette-Guérin (BCG, *Mycobacterium bovis* vaccine) on reducing SARS-CoV-2 infection, by inducing a trained innate immune response [20,21]; this trained immunity is a non-specific response activated through epigenetic changes in myeloid cells and NK cells that lead to a long-term proinflammatory response and confers cross-protection against other pathogens [22]. Still, the development and administration of SARS-CoV-2 specific vaccines is crucial for limiting the COVID-19 pandemic [23,24,25], while the previous ones could be a complement to SARS-CoV-2 specific vaccines in certain settings until herd immunity is achieved with the latest.

One of the major limitations of our study was the small sample size of patients vaccinated for only Influenza and their characteristics; some of these patients had respiratory comorbidities or other factors in their medical history (not collected here) that recommended the Influenza vaccination. They might have received the vaccine before pregnancy or during the first weeks of pregnancy. The Influenza vaccination campaign (1 October 2019 to 31 January 2020) was prior to COVID-19 lockdown measures in our country, a situation that may have conditioned the subsequent Tdap vaccination (as Tdap vaccine is administered from the 28th week of gestation onwards). Furthermore, the percentage of premature births in this group (5/23, 21.7%) was higher than the observed in the remaining three groups and we should consider that they may have given birth before getting the vaccine. 

However, the unusually high proportion of respiratory comorbidities as well as COVID-19 symptoms and their severity in the Influenza vaccine group, is possibly a statistical anomaly due to the small sample size of this group. If there really was an association between the Influenza vaccine and a worse prognosis of the disease, this would have also been seen in the group that received both vaccines (Tdap and Influenza). If this had been the case, we would have had to consider running a multivariable analysis, adjusting for the presence of respiratory comorbidities in these patients. 

Another limitation of our study was the lack of information about the exact dates when these vaccines were administered or, in case of multiparous women, if the vaccines were administered in previous pregnancies.

Focusing on the sociodemographic characteristics of the patients, our study supports the previously reported higher risk of poor evolution of the SARS-CoV-2 infection in Latin-Americans [8]. We cannot attribute this prognosis to the vaccination status of these patients and, in the absence of plausible genetic differences, it should be considered that these subjects may be less inclined to follow other preventive measures established for infectious disease control [26]. The objective of this study was not to assess whether there may be a conscious rejection or a difficulty/impossibility of compliance, although we suggest that these findings should be considered in preventive and public health policies [27].

Since differences were not observed in the clinical evolution of SARS-CoV-2 infection in pregnant patients complying with the current vaccination programs, we support the government’s recommendation for massive seasonal vaccination against Influenza, and especially for pregnant women, in order to avoid clinical complications in these patients [28,29]. Health education campaigns should be specially targeted to groups less likely to participate in vaccination programs, as well as for a future SARS-CoV-2 vaccination campaign [30].

## 5. Conclusions

No association was observed between the Influenza and/or Tdap vaccination status of patients in the current pregnancy and the clinical presentation of SARS-CoV-2 infection or the severity of symptoms. Adherence to vaccination was observed to be ethnicity dependent; therefore, health education campaigns should be specially targeted to these groups.

## Figures and Tables

**Table 1:** Baseline characteristics of the study participants and by vaccination group.

Baseline Characteristics	Total *N* = 1150	Vaccination Groups				
		No Vaccine *N* = 183	Influenza Alone *N* = 23	Tdap Alone *N* = 529	Both Vaccines *N* = 415	*p*-Value
Maternal Age (Years; Median/IQR)	33 (28–37)	32 (26–36)	33 (26–39)	33 (28–36)	33 (29–37)	0.013 *
Ethnicity						0.001 *
Latin-American	308/1147 (26.9)	48 (26.2)	7 (30.4)	172/527 (32.6)	81/414 (19.6)	
Caucasian	674/1147 (58.8)	107 (58.5)	14 (60.9)	280/527 (53.1)	273/414 (65.9)	
Other ^a^	165/1147 (14.4)	28 (15.3)	2 (8.7)	75/527 (14.2)	60/414 (14.5)	
Private hospital	83 (7.2)	14 (7.7)	0 (0.0)	31 (5.9)	38 (9.2)	0.131
Nulliparity	455 (39.6)	78 (42.6)	12 (52.2)	206 (38.9)	159 (38.3)	0.457
Preterm delivery (<37 weeks)	113/1148 (9.8)	29 (15.8)	5 (21.7)	37 (7.0)	42/413 (10.2)	0.001 *
In vitro fertilization	69 (6.0)	7 (3.8)	0 (0.0)	26 (4.9)	36 (8.7)	0.025 *
Respiratory comorbidities	44 (3.8)	8 (4.4)	4 (17.4)	17 (3.2)	15 (3.6)	0.007 *
Chronic lung disease	3 (0.3)	1 (0.5)	0 (0.0)	1 (0.2)	1 (0.2)	0.863
Asthma	43 (3.7)	8 (4.4)	4 (17.4)	17 (3.2)	14 (3.4)	0.005 *

Data are shown as n (% of total with data), except where otherwise indicated. IQR: Interquartile Range ^a^ Arab, Black, East-Asian, South-Asian and West-Asian * Statistically significant differences.

**Table 2:** Clinical presentation of SARS-CoV-2 infection by vaccination group.

Clinical Presentation of SARS-CoV-2 infection	No Vaccine *N* = 183	Influenza Alone *N* = 23	Tdap Alone *N* = 529	Both Vaccines *N* = 415	*p*-Value
Asymptomatic	85 (46.4)	6 (26.1)	276 (52.2)	217 (52.3)	0.051
Symptomatic	98 (53.6)	17 (73.9)	253 (47.8)	198 (47.7)	
Mild-moderate symptoms	70/98 (71.4)	10/17 (58.8)	182/253 (71.9)	147/198 (74.2)	0.433
Pneumonia	22/98 (22.4)	5/17 (29.4)	62/253 (24.5)	46/198 (23.2)	
Complicated pneumonia ^a^/shock	6/98 (6.1)	2/17 (11.8)	9/253 (3.6)	5/198 (2.5)	

Data are shown as n (% of total); ^a^ with ICU admission and/or mechanical ventilation and/or septic shock.

**Table 3:** Baseline characteristics by clinical presentation of SARS-CoV-2 infection.

Baseline Characteristics	All Patients *N* = 1150			Symptomatic Patients *N* = 566			
	Asymptomatic Patients *N* = 584	Symptomatic Patients *N* = 566	*p*-Value	Mild–Moderate Symptoms *N* = 409	Pneumonia *N* = 135	Complicated Pneumonia ^c^/Shock *N* = 22	*p*-Value
Maternal Age (Years; Median/IQR)	32 (28–36)	33 (28–37)	0.153	33 (28–37)	33 (28–37)	32 (26–38)	0.848
Ethnicity			<0.001 *				0.032 *
Latin-American	106/583 (18.2)	202/564 (35.8)		131/407 (32.2)	63 (46.7)	8 (36.4)	
Caucasian	375/583 (64.3)	299/564 (53.0)		231/407 (56.8)	56 (41.5)	12 (54.5)	
Other ^a^	102/583 (17.5)	63/564 (11.2)		45/407 (11.1)	16 (11.9)	2 (9.1)	
Private hospital	58 (9.9)	25 (4.4)	<0.001 *	19 (4.6)	6 (4.4)	0 (0.0)	0.586
Nulliparity	235 (40.2)	220 (38.9)	0.635	167 (40.8)	46 (34.1)	7 (31.8)	0.297
In vitro fertilization	31 (5.3)	38 (6.7)	0.316	30 (7.3)	5 (3.7)	3 (13.6)	0.143
Respiratory comorbidities	15 (2.6)	29 (5.1)	0.024 *	22 (5.4)	5 (3.7)	2 (9.1)	0.515
Chronic lung disease	1 (0.2)	2 (0.4)	0.619	0 (0.0)	2 (1.5)	0 (0.0)	0.041 *
Asthma	15 (2.6)	28 (4.9)	0.034 *	22 (5.4)	4 (3.0)	2 (9.1)	0.351

Data are shown as n (% of total with data), except where otherwise indicated; IQR: Interquartile Range; ^a^ Arab, Black, East-Asian, South-Asian and West-Asian; ^c^ with ICU admission and/or mechanical ventilation and/or septic shock; * Statistically significant differences.

## Data Availability

Restrictions apply to the availability of these data. Data belongs to the Institute of Health Carlos III and the Spanish Ministry of Health, and are available from the authors with the permission of the Institute of Health Carlos III and the Spanish Ministry of Health.

## Supplementary Materials

The following are available online at https://www.mdpi.com/2076-393X/9/1/31/s1. Table S1: List of hospitals included in the study (*n* = 78).

## References

[1] Jehi L., Ji X., Milinovich A., Erzurum S., Merlino A., Gordon S., Young J.B., Kattan M.W. (2020). Development and validation of a model for individualized prediction of hospitalization risk in 4536 patients with COVID-19. PLoS ONE.

[2] Murillo-Zamora E., Trujillo X., Huerta M., Ríos-Silva M., Mendoza-Cano O. (2020). Male gender and kidney illness associated with an increased risk of severe laboratory-confirmed coronavirus disease. BMC Infect. Dis..

[3] Ortiz-Prado E., Simbana-Rivera K., Diaz A.M., Barreto A., Moyano C., Arcos V., Vasconez-Gonzalez E., Paz C., Simbana-Guaycha F., Molestina-Luzuriaga M. (2020). Epidemiological, Socio-Demographic and Clinical Features of the Early Phase of the COVID-19 Epidemic in Ecuador. medRxiv.

[4] Poblador-Plou B., Carmona-Pírez J., Ioakeim-Skoufa I., Poncel-Falcó A., Bliek-Bueno K., Cano-Del Pozo M., Gimeno-Feliú L.A., González-Rubio F., Aza-Pascual-Salcedo M., Bandrés-Liso A.C. (2020). Baseline Chronic Comorbidity and Mortality in Laboratory-Confirmed COVID-19 Cases: Results from the PRECOVID Study in Spain. Int. J. Environ. Res. Public Health.

[5] Fink G., Orlova-Fink N., Schindler T., Grisi S., Ferrer A.P., Daubenberger C., Brentani A. (2020). Inactivated Trivalent Influenza Vaccine Is Associated with Lower Mortality among Covid-19 Patients in Brazil. medRxiv.

[6] Reche P.A. (2020). Potential Cross-Reactive Immunity to SARS-CoV-2 From Common Human Pathogens and Vaccines. Front. Immunol..

[7] Forster A.S., Rockliffe L., Chorley A.J., Marlow L.A.V., Bedford H., Smith S.G., Waller J. (2017). Ethnicity-specific factors influencing childhood immunisation decisions among Black and Asian Minority Ethnic groups in the UK: A systematic review of qualitative research. J. Epidemiol. Community Health.

[8] Ellington S., Strid P., Tong V.T., Woodworth K., Galang R.R., Zambrano L.D., Nahabedian J., Anderson K., Gilboa S.M. (2020). Characteristics of Women of Reproductive Age with Laboratory-Confirmed SARS-CoV-2 Infection by Pregnancy Status—United States, January 22–June 7, 2020. MMWR Morb. Mortal. Wkly Rep..

[9] Pardilla M.B.E., Aguilar A.C., Puig B.M., Lorenzana A.S., de la Torre I.R., de la Manzanara P.H.L., Bernardo A.F., Pérez Ó.M. (2020). Spanish registry of Covid-19 screening in asymptomatic pregnants. Rev. Esp. Salud. Publica..

[10] Ministerio de Sanidad, Consumo y Bienestar Social, Gobierno de España Preguntas y Respuestas Sobre la Vacunación Frente a la Gripe 2020–2021. https://www.mscbs.gob.es/profesionales/saludPublica/prevPromocion/vacunaciones/programasDeVacunacion/gripe/faq/Preguntas_respuestas_gripe_ciudadanos_2020-2021.htm.

[11] Ministerio de Sanidad, Servicios Sociales e Igualdad, Gobierno de España Preguntas y Respuestas sobre la Vacunación de la Tosferina en Embarazadas. https://www.mscbs.gob.es/ciudadanos/proteccionSalud/vacunaciones/docs/Vacunacion_Tosferina_Embarazadas.pdf.

[12] WHO Clinical Management of COVID-19. Interim Guidance 27 May 2020. WHO/2019-nCoV/clinical/2020.5. https://www.who.int/publications/i/item/clinical-management-of-covid-19.

[13] Bates D., Maechler M., Bolker B., Walker S. (2015). Fitting Linear Mixed-Effects Models Using lme4. J. Stat. Soft..

[14] WHO (2013). Global Vaccine Action Plan 2011–2020.

[15] Ministerio de Sanidad, Consumo y Bienestar Social, Gobierno de España Coberturas de Vacunación, Datos Estadísticos (Actualización Enero 2020). https://www.mscbs.gob.es/profesionales/saludPublica/prevPromocion/vacunaciones/calendario-y-coberturas/coberturas/home.htm.

[16] Lu P.J., O’Halloran A., Bryan L., Kennedy E.D., Ding H., Graitcer S.B., Santibanez T.A., Meghani A., Singleton J.A. (2014). Trends in racial/ethnic disparities in influenza vaccination coverage among adults during the 2007-08 through 2011-12 seasons. Am. J. Infect. Control..

[17] Hoogink J., Verelst F., Kessels R., Jan van Hoek A., Timen A., Willem L., Beutels P., Wallinga J., de Wit G.A. (2020). Preferential differences in vaccination decision-making for oneself or one’s child in The Netherlands: A discrete choice experiment. BMC Public Health.

[18] Verelst F., Kessels R., Delva W., Beutels P., Willem L. (2019). Drivers of vaccine decision-making in South Africa: A discrete choice experiment. Vaccine.

[19] Martínez-Baz I., Trobajo-Sanmartín C., Arregui I., Navascués A., Adelantado M., Indurain J., Fresán U., Ezpeleta C., Castilla J. (2020). Influenza Vaccination and Risk of SARS-CoV-2 Infection in a Cohort of Health Workers. Vaccines.

[20] Berg M.K., Yu Q., Salvador C.E., Melani I., Kitayama S. (2020). Mandated Bacillus Calmette-Guérin (BCG) Vaccination Predicts Flattened Curves for the Spread of COVID-19. medRxiv.

[21] Miller A., Reandelar M.J., Fasciglione K., Roumenova V., Li Y., Otazu G.H. (2020). Correlation between Universal BCG Vaccination Policy and Reduced Mortality for COVID-19. medRxiv.

[22] Netea M.G., Joosten L.A.B., Latz E., Mills K.H.G., Natoli G., Stunnenberg H.G., ONeill L.A.J., Xavier R.J. (2016). Trained Immunity: A Program of Innate Immune Memory in Health and Disease. Science.

[23] Huang J., Huang H., Wang D., Wang C., Wang Y. (2020). Immunological strategies against spike protein: Neutralizing antibodies and vaccine development for COVID-19. Clin. Transl. Med..

[24] COVID-19 Real-Time Learning Network. Vaccines in Development (Last Updated: 24 December 2020). https://www.idsociety.org/covid-19-real-time-learning-network/vaccines/vaccines/.

[25] Polack F.P., Thomas S.J., Kitchin N., on behalf of the C4591001 Clinical Trial Group (2020). Safety and Efficacy of the BNT162b2 mRNA Covid-19 Vaccine. N. Engl. J. Med..

[26] How to Protect Yourself and Others. https://www.cdc.gov/coronavirus/2019-ncov/prevent-getting-sick/prevention.html.

[27] Thomson A., Vallée-Tourangeau G., Suggs L.S. (2018). Strategies to increase vaccine acceptance and uptake: From behavioral insights to context-specific, culturally-appropriate, evidence-based communications and interventions. Vaccine.

[28] Grohskopf L.A., Sokolow L.Z., Broder K.R., Olsen S.J., Karron R.A., Jernigan D.B., Bresee J.S. (2016). Prevention and control of seasonal influenza with vaccines: Recommendations of the Advisory Committee on Immunization Practices–United States, 2016-17 influenza season. MMWR Recomm. Rep..

[29] Poehling K.A., Edwards K.M., Weinberg G.A., Szilagyi P., Staat M.A., Iwane M.K., Bridges C.B., Grijalva C.G., Zhu Y., Bernstein D.I. (2006). The underrecognized burden of influenza in young children. N. Engl. J. Med..

[30] Doherty M., Schmidt-Ott R., Santos J.I., Stanberry L.R., Hofstetter A.M., Rosenthal S.L., Cunningham A.L. (2016). Vaccination of special populations: Protecting the vulnerable. Vaccine.

